# Crystal Structure of Venezuelan Hemorrhagic Fever Virus Fusion Glycoprotein Reveals a Class 1 Postfusion Architecture with Extensive Glycosylation

**DOI:** 10.1128/JVI.02298-13

**Published:** 2013-12

**Authors:** Marie-Laure Parsy, Karl Harlos, Juha T. Huiskonen, Thomas A. Bowden

**Affiliations:** Division of Structural Biology, Wellcome Trust Centre for Human Genetics, University of Oxford, Oxford, United Kingdom

## Abstract

Guanarito virus (GTOV) is an emergent and deadly pathogen. We present the crystal structure of the glycosylated GTOV fusion glycoprotein to 4.1-Å resolution in the postfusion conformation. Our structure reveals a classical six-helix bundle and presents direct verification that New World arenaviruses exhibit class I viral membrane fusion machinery. The structure provides visualization of an N-linked glycocalyx coat, and consideration of glycan dynamics reveals extensive coverage of the underlying protein surface, following virus-host membrane fusion.

## TEXT

Guanarito virus (GTOV), first recognized in 1989, is the etiological agent of Venezuelan hemorrhagic fever (1). Human transmission of this highly virulent virus from rodent reservoirs probably occurs primarily through contamination of food stocks and dust particles (2, 3). Clinical manifestations include fever and malaise, which develop into hemorrhagic fever (HF) (4). As a result of the rapid onset and high mortality rates associated with infection (∼23%) (2), GTOV is classified as a high-priority category A biothreat agent (5). Together with other South American HF arenaviruses, such as Machupo virus (MACV), Junin virus (JUNV), and Sabia virus (SABV), GTOV belongs to the Tacaribe complex of the New World clade of the *Arenaviridae* family (6).

The GTOV genome encodes two highly glycosylated glycoproteins which facilitate host cell attachment (GP1) and fusion (GP2). These are primary targets for antiviral drug design (7–9). GP1 binds transferrin receptor 1 (TfR1) with high affinity (10–16). Based upon sequence homology with the structure of the Old World lymphocytic choriomeningitis virus (LCMV) fusion glycoprotein, GP2 has been postulated to form a class I fusion fold (17). Upon acidification during clatherin-mediated endocytosis, GP1 is released from the virion and GP2 mediates fusion of the viral and host membranes (18–22). While GP1 sequences are varied (30 to 47% sequence identity) among New World arenaviruses, GP2 sequences are very similar (60 to 70% identity), indicating conserved function and structure (23, 24).

The globular architecture and mode of receptor recognition by New World GP1 is established (10, 12). However, despite the biomedical impact of New World arenaviruses, there are no crystallographic data describing the structure of the GP2 fusion glycoprotein. Here, we broaden the structural coverage of the mature New World glycoprotein subunits through analysis of GTOV GP2.

**Fig 1:**
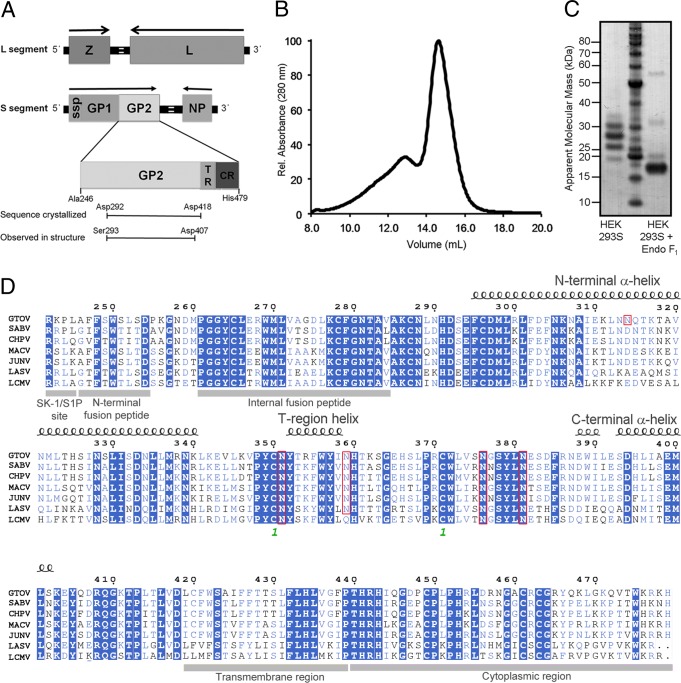
Construct design, purification, and sequence analysis of GTOV GP2. (A) Schematic diagram of the arenavirus genome, with details of the construct design of the GTOV structure. Arenaviruses contain a bisegmented, ambisense RNA genome. The long RNA segment encodes the RNA polymerase (L) and matrix protein (Z). The short segment encodes the nucleoprotein (NP) and a glycoprotein precursor (GPC). Proteolytic cleavage of GPC by the cellular proprotein convertase site 1 protease (SK-1/S1P) (45) yields three products: a stable signal peptide (ssp) required for maturation (46–48), GP1, and GP2. These components noncovalently associate to form the GP, which further assembles into a trimeric spike on the virion surface (49). TR, transmembrane region; CR, cytoplasmic region. (B) Size exclusion chromatogram of glycosylated, trimeric GTOV GP2 run on a Superdex 200 10/30 column (Amersham) equilibrated in 150 mM NaCl and 10 mM Tris, pH 8.0. Protein yields were approximately 3.0 mg purified protein per liter cell culture. (C) The results of an SDS-PAGE assay run under reducing conditions show the glycosylated GTOV GP2 (left lane), a molecular mass marker (center lane), and GTOV GP2 deglycosylated with endoglycosidase F1 (Endo F_1_) (right lane). The expected unglycosylated molecular mass of the deglycosylated GTOV protomer is approximately 18 kDa. (D) Structure-based sequence alignment, plotted with ESPript (50), of GTOV GP2 with the GP2s of SABV, Chapare virus (CHPV), MACV, JUNV, Lassa virus (LASV), and LCMV. α-Helices are shown as spirals. Residues which are highlighted in blue are fully conserved, residues which are colored blue are partially conserved, and residues which are black are not conserved. Residues Cys350 and Cys371 participate in a disulfide bond and are denoted with a green number 1. Amino acids which correspond to predicted N-linked glycosylation sites are marked with red boxes.

GTOV GP2 (GenBank accession number AAS55656.1, residues 292 to 418) was cloned into the pOPINTTGNeo mammalian expression vector (25). Approximately 50 residues at the N terminus of the GTOV GP2 ectodomain were removed to exclude the aggregation-inducing N-terminal hydrophobic fusion loop (Fig. 1A). To aid crystallogenesis, recombinant glycoprotein expression was performed in GlcNAc transferase I-deficient human embryonic kidney 293S (HEK 293S) cells, which traps glycosylation predominantly to a homogenous Man_5_GlcNAc_2_ glycoform (26–28). Transiently expressed GTOV GP2 was purified using immobilized metal affinity chromatography followed by size exclusion chromatography (Fig. 1B). SDS-PAGE analysis of purified protein revealed multiple bands, separated in mass by approximately 2 kDa. These bands likely correspond to differential occupancies of N-linked glycosylation (Fig. 1C). GTOV GP2 was concentrated to 7.5 mg/ml and crystallized using the sitting-drop vapor diffusion method of 100 nl protein plus 100 nl precipitant (0.15 M Li_2_SO_4_, 0.1 M citric acid, pH 3.5, 18% [wt/vol] polyethylene glycol 6000 [PEG 6000]) equilibrated against 95-μl reservoirs at 22**°**C (29). Crystals were immersed in reservoir solution containing 25% (vol/vol) ethylene glycol before being cooled to 100 K. X-ray diffraction data were recorded at beamline I04, Diamond Light Source, to 4.1-Å resolution. Images were indexed, integrated, and scaled with HKL2000 (Table 1) (30).

**Table 1:** Crystallographic data and refinement statistics^*g*^

Parameter	Result
Data collection statistics	
Resolution range (Å)	50.0–4.14 (4.29–4.14)^*a*^
Space group	*P*4_1_
Cell dimensions	
*a*, *b*, *c* (Å)	99.2, 99.2, 79.9
α, β, γ (°)	90.0, 90.0, 90.0
Wavelength (Å)	0.953
No. of unique reflections	5,894 (576)^*a*^
Completeness (%)	100.0 (99.6)
*R*_merge_ (%)^*b*^	9.9 (>100.0)
*I*/σ*I*	26.6 (1.9)
Avg redundancy	5.1 (5.1)
CC_1/2_^*c*^	0.998 (0.362)
Refinement statistics	
Resolution range (Å)	38.4–4.14 (4.63–4.14)^*a*^
No. of reflections	5,561 (1,563)^*a*^
*R*_work_ (%)^*d*^	25.5 (29.1)
*R*_free_ (%)^*e*^	27.6 (33.7)
RMSD	
Bonds (Å)	0.010
Angle (°)	1.4
Between NCS-related Cα atoms	0.8
No. of molecules per ASU	3
No. of atoms per ASU (protein/carbohydrate)	2,649/358
Avg *B* factors (Å^2^) (protein/carbohydrate)	214/290
Model quality (Ramachandran plot)^*f*^	
Favored region (%)	91.2
Allowed region (%)	95.5
Disallowed region (%)	4.5

a Numbers in parentheses refer to the relevant outer resolution shell.

b *R*_merge_ = Σ_hkl_ Σ_i_|*I*(*hkl;i*) − <*I*(*hkl*)>|/Σ_hkl_ Σ_i_*I*(*hkl*;*i*), where *I*(*hkl*;*i*) is the intensity of an individual measurement and <*I*(*hkl*)> is the average intensity from multiple observations.

c CC_1/2_ is defined in reference 51.

d *R*_work_ = Σ_hkl_‖*F*_obs_| −*k*|*F*_calc_‖/Σ_hkl_ |*F*_obs_|.

e *R*_free_ is calculated as for *R*_work_, but using only 5% of the data which were sequestered prior to refinement.

f Ramachandran plots were calculated with MolProbity (34).

g Data were obtained at the Diamond I04 beamline. ASU, asymmetric unit; NCS, noncrystallographic symmetry.

The structure was solved by molecular replacement with Phaser (31) using aglycosylated LCMV GP2 as a search model (PDB accession number 3MKO [17]). Model building was performed with Coot (32). The crystal contained protein-glycan and glycan-glycan lattice contacts, which facilitated visualization of carbohydrate chains from three out of the five N-linked glycosylation sites on GP2. The two remaining N-linked sites, although likely to be at least partially occupied (Fig. 1C), lacked surrounding stabilizing environments and were thus not visible in the crystal structure. Oligomannose-type glycans were built based on carbohydrate from PDB accession number 2WAH (33). Model building was facilitated by map sharpening (35). Refinement in Buster (36) used local structural similarity restraints (LSSR) to the high-resolution 3MKO structure, grouped B-factor refinement (grouped by chain), tensor libration screw (TLS) modeling, and local 3-fold noncrystallographic symmetry restraints (Table 1). The final protein structure was validated using MolProbity (34).

**Fig 2:**
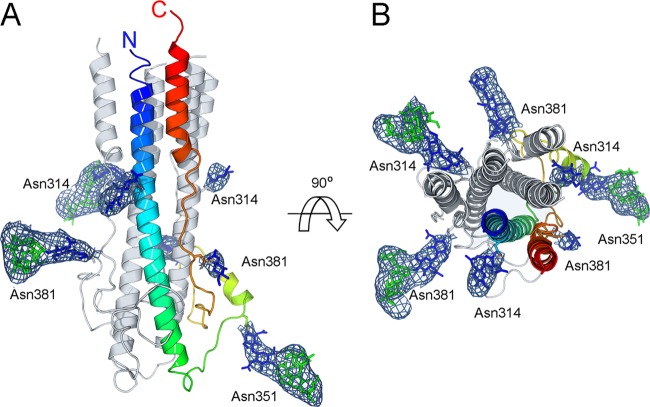
Structure of glycosylated GTOV GP2. (A) Cartoon diagram of trimeric GTOV GP2 in the postfusion conformation. One protomer in the asymmetric unit is colored as a rainbow, with the N terminus shown in blue and the C terminus in red. N-linked carbohydrates are shown as sticks, with GlcNAc residues colored blue and mannose residues colored green. A maximum-likelihood weighted 2*F*_o_−*F*_c_ electron density map is plotted around each glycan at 1σ. (B) View of panel A rotated by 90°.

**Fig 3:**
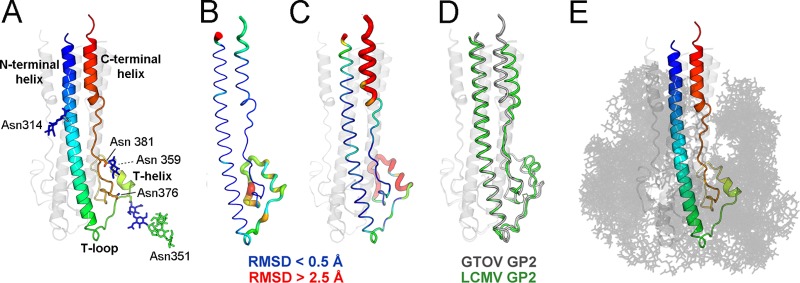
Structural plasticity and the glycocalyx, which coats GTOV GP2. (A) Single protomer of GTOV, colored as described for Fig. 2. (B) GTOV protomer with root mean square (RMS) displacement of equivalent residues between protomers mapped onto the Cα trace. The tube radius and color represent the RMS displacement (ramped from blue to red). Regions with high deviations between protomers are thick and red. Regions with low deviations are thin and blue. (C) RMS displacement of equivalent residues between GTOV GP2 (chain B, the best-ordered protomer of the trimer) and LCMV GP2. The color scheme matches that of panel B. (D) Cα trace of GTOV GP2 (gray) superimposed with LCMV (green) (PDB accession number 3MKO). (E) GTOV GP2, illustrated as described for panel A, with glycans (opaque gray lines) modeled at each N-linked sequon observed in the structure. Multiple orientations of each glycan are shown to illustrate carbohydrate conformational flexibility and how this results in occlusion of the GTOV GP2 protein surface.

Each of the three GP2 subunits in the asymmetric unit consists of three regions: a 45-amino-acid N-terminal α-helix (residues 301 to 346), a 50-amino-acid “T region” (residues 347 to 398; named in comparison with the Old World LCMV GP2 structure [17]), and a short C-terminal α-helix (residues 399 to 408) (Fig. 2A and B and 3A and B). The N-terminal α-helix spans the entire length of the 85-Å-long molecule and connects to the C-terminal helix through the T region, which is composed of loops and a small α-helix (residues 358 to 364). The N- and C-terminal helices pack closely in an antiparallel arrangement, where each protomer associates to form a trimeric coiled coil. The N-terminal part of this coiled coil contains the previously described “stutter” structure, which is common throughout class I viral fusion proteins (17). This confirms that the New World GP2 is a class I fusion glycoprotein. Our structure is in a postfusion conformation (8, 37), with the N terminus (fusion loop region) and C terminus (transmembrane region) colocalized, consistent with the merger of the virion and host cell membranes (Fig. 2).

The closest relative of GTOV GP2 with a known structure is aglycosylated Old World LCMV GP2 (17). Both structures are identical in length, exhibit very similar secondary structures, and maintain approximately 50% amino acid sequence identity (Fig. 3C and D). However, superposition of equivalent GTOV and LCMV protomers leads to a greater structural variation between the two structures (a 2.2-Å root mean square deviation [RMSD] over 96 Cα residues) than expected between two structures with 50% sequence identity (an ∼1.0-Å RMSD [24]). This is largely due to rigid-body translational differences in the organization of the three regions (Fig. 3C and D). While the N-terminal helices of GTOV and LCMV independently superpose well (1.05-Å RMSD over 50 Cα residues), the locations of many equivalent residues in the T region and C-terminal helices differ by more than 3 Å (Fig. 3D). Such structural dissimilarities may reflect intrinsic differences between Old and New World architectures.

In contrast to the well-ordered N- and C-terminal helical regions, the T region exhibits a high degree of flexibility. Comparison of the three independent GTOV protomers reveals that there are differences between loop conformations in the T region (Fig. 3B). The T region also contains a 10-amino-acid stretch (residues 365 to 375) which is fully ordered in one protomer and disordered in the second and third. Differences in T regions are likely due to differential crystal packing environments of individual protomers. Surprisingly, the T region does not correlate with marked sequence divergence (Fig. 1D) (60% of T-region residues are conserved among New World arenaviruses).

Our structure was determined in the absence of the N-terminal 50-amino-acid segment, corresponding to the bipartite fusion loop. Although it would be interesting to investigate how this loop is structured and how it might influence the flexibility of the underlying T regions, this peptide is orientated in the opposite direction, toward the target host membrane (Fig. 1). We therefore predict limited direct interactions between these regions.

N-linked glycosylation plays a key role in arenavirus glycoprotein folding, maturation, cellular tropism, viral fusion, and immune evasion (38–40). As estimated by SDS-PAGE analysis, N-linked glycosylation contributes up to 40% of the mass of recombinant GTOV GP2 (Fig. 1C) (28). The N-linked sequons are differentially occupied when recombinantly expressed, supporting the conclusion that most of these sites are, at least individually, dispensable for folding (Fig. 1C). Analysis by the NetNGlyc server (http://www.cbs.dtu.dk/services/NetNGlyc/), which is trained on a database of experimentally determined glycan occupancy data (41), supports the conclusion that GTOV GP2 likely contains partially occupied sites on the native infectious virion.

A high density of carbohydrate covers the surface of the GP2 postfusion structure. Based on the visible electron density of these sites (Fig. 2), we created a model for how N-linked glycosylation is presented by GP2 following fusion (Fig. 3E). Although we do not preclude the presence of oligomannose- or hybrid-type structures on the mature virion, this was performed using the structure of a complex glycan (42), typical of secreted glycoproteins (43), as opposed to the artificial glycosylation structure engineered in this study. Consideration of the natural conformational flexibility of these glycans (44), when not restricted by crystallographic packing, reveals that the majority of the protein surface is occluded by a dynamic glycocalyx, with only the N- and C-terminal membrane-proximal regions readily accessible (Fig. 3E).

Unlike the glycosylation sites on GP1 (12), the sites on GP2 are well conserved across New World arenaviruses, with the exception of one unique site in GTOV (Asn314) (Fig. 1D). The conservation of all but one site suggests that there is selective pressure to maintain these positions. This restriction is likely to be driven, in part, by the requirement of GP2 glycosylation sites to be solvent accessible in both prefusion (GP1-bound [18, 19]) and postfusion states, a factor that does not significantly influence GP1. Because of the sheer density of glycans on GP2 and the requirement for association with GP1 (18, 19), we suggest that ordering some of the glycans may be necessary for the productive formation of the GP1-GP2 heterodimer (Fig. 3E). A further consequence of this hypothesis might be that the glycans may be less processed than predicted due to steric constraints of the overall packing environment of the GP-GP2 heterotrimer. Given the influence of carbohydrate processing on arenaviral tropism and immune responses (38–40), it would be valuable to know the carbohydrate compositions of native virions.

We present here the first structure of a New World arenavirus fusion glycoprotein and show that GTOV GP2 adopts an archetypal class I-type postfusion fold. The positions of the well-ordered N- and C-terminal helical regions differ from those of the equivalent Old World LCMV, and our structure thereby provides an improved template for the New World fusion glycoprotein. In our structure, we also visualize N-linked carbohydrates that form a glycocalyx and obscure much of the protein surface. On the whole, our analysis broadens the structural coverage of the mature New World glycoprotein subcomponents and provides evidence that arenaviral glycoprotein architecture can vary between clades.

### Protein structure accession number.

Coordinates and structure factors have been deposited in the Protein Data Bank (accession number 4C53).
